# Temporal trends in transfusion-transmissible infections among blood donors in Jordan: A large-scale Amman-based surveillance update (2018–2019)

**DOI:** 10.1097/MD.0000000000045847

**Published:** 2025-11-21

**Authors:** Ali Atoom, Baheieh Al-Abadi, Mansour Al-Hiary, Bayan Alzubi, Sanaa Al-Saeidat, Talal Al-Qaisi

**Affiliations:** a Department of Medical Laboratory Sciences, Al-Ahliyya Amman University, Amman, Jordan; b Pharmacological and Diagnostic Research Center (PDRC), Al-Ahliyya Amman University, Amman, Jordan; c Princess Iman Center for Research and Laboratory Sciences, Jordanian Royal Medical Services, Amman, Jordan; d Department of Medical Laboratory Sciences, Al-Ahliyya Amman University, Amman, Jordan; e Hematology Department, Prince Iman Center, Amman, Jordan; f Department of Biomedical Sciences, College of Health Sciences, Abu Dhabi University, Abu Dhabi, United Arab Emirates.

**Keywords:** blood donors, blood safety, hepatitis B, hepatitis C, HIV infections, transfusion-transmitted infection

## Abstract

Transfusion-transmissible infections (TTIs) remain a global concern in blood safety. This study presents the largest Amman-based dataset on Royal Medical Services donors since 2016 (n = 49,296 donations) and the first to apply a monthly Chi-square (χ^2^) trend analysis for TTI surveillance in Jordan. Blood donations collected between July 2018 and December 2019 were screened for hepatitis B surface antigen (HBsAg), hepatitis B core antibody (HBcAb), hepatitis C virus antibody (HCV Ab), human immunodeficiency virus (HIV) 1/2 antibodies, and *Treponema pallidum* using electrochemiluminescence assays, followed by confirmatory enzyme-linked immunosorbent assay tests. HBcAb was detected in 3.44% of donors, HBsAg in 0.41%, and HCV Ab in 0.10%. No confirmed cases of HIV or syphilis were found. These rates are lower than previously reported national estimates, suggesting a continued decline in hepatitis B virus and hepatitis C virus (HCV) prevalence. Chi-square tests revealed significant monthly variation for HBcAb (*P* < .001) and HBsAg (*P* < .001), while HCV trends were not significant. The majority of donors were male (98.2%) and from Amman (98.24%), limiting national generalizability. Although the dataset predates the COVID-19 pandemic and is geographically constrained, it remains the most recent and comprehensive post-2016 dataset available for national TTI surveillance. This study highlights low national TTI prevalence and underscores the value of blood donor screening as a tool for public health surveillance, especially in refugee-hosting contexts. The application of temporal analysis enhances its relevance for infection monitoring and health policy. Findings support the integration of seasonal trend surveillance into Jordan’s national blood safety strategy and align with global goals to eliminate communicable diseases by 2030.

## 1. Introduction

Blood transfusion remains a critical, life-saving medical intervention, but it carries the risk of transmitting blood-borne pathogens collectively referred to as transfusion-transmitted infections (TTIs). These infections include hepatitis B virus (HBV), hepatitis C virus (HCV), human immunodeficiency virus (HIV), and *Treponema pallidum.*^[1]^ To ensure the safety of blood supplies, the World Health Organization (WHO) recommends universal screening of donated blood using sensitive and specific assays.^[2]^ Despite these efforts, disparities persist: although high-income countries collect around 40% of global blood donations, they account for only 16% of the world’s population. In contrast, low- and middle-income countries, including many in the Eastern Mediterranean region, rely heavily on transfusions for vulnerable populations such as children under 5, with 54% of transfusions going to this age group.^[3]^

Effective laboratory screening and strict donor eligibility criteria are essential to minimizing the transmission of TTIs and maintaining transfusion safety.^[4]^ Blood donor screening data play a crucial role in public health surveillance by providing valuable insights into the prevalence of infectious diseases within the general population.^[5]^ In line with the WHO’s 2030 goal of eliminating viral hepatitis and HIV as major public health threats, this target as a 90% reduction in new infections and a 65% reduction in related deaths from 2015 levels. Accurate and updated epidemiological data are vital for guiding policy and prevention programs.^[6]^ Therefore, countries must obtain comprehensive prevalence data regarding TTI from blood donors, as healthy blood donors may reflect the general population status; thus, screening data from this group is considered a prerequisite baseline in implementing preventive control strategies towards better public health settings.^[7]^

The sustainable development goals (SDG) emphasize the importance of inclusive, disaggregated health data to track infectious disease trends, especially in host countries with large displaced populations such as Jordan.^[8]^ Furthermore, The sustainable development goals (SDG 3.3) call for ending the epidemics of HIV, hepatitis, and other communicable diseases by 2030. Achieving this requires inclusive, disaggregated health data from all populations, especially in host countries like Jordan that face complex demographic challenges.^[9]^ This is the largest TTI dataset analyzed in Jordan to date, encompassing nearly 50,000 donor records. This study provides the most recent post-2016, large-scale surveillance TTIs among Jordanian blood donors, offering critical epidemiological data aligned with WHO’s 2030 elimination targets. The study contributes timely insight into infection trends amid regional demographic shifts, leveraging donor screening as a proxy for public health surveillance.

Previous national data on transfusion-transmitted infections (TTIs) in Jordanindicated prevalence rates of 0.52% for hepatitis B surface antigen (HBsAg), 6.04% for hepatitis B core antibody (HBcAb), and 0.16% for HCV antibodies among healthy blood donors, with no cases of HIV detected. While these findings provided a valuable baseline, they reflect data collected between 2009 and 2013. They may no longer reflect current infection patterns amid new public health and demographic pressures.^[10]^

More recent national studies have since been conducted. Blood donors from 2016 to 2019 at the National Blood Bank in Amman, reporting similar TTI prevalence rates: 0.52% for HBsAg, 6.04% for HBcAb, and 0.16% for hepatitis C virus antibody (HCV Ab).^[11]^ A larger national dataset (n = 31,014) from 2014 to 2019, with findings of 0.50% HBsAg, 5.74% HBcAb, and 0.14% HCV Ab, also reporting 0.01% syphilis and no confirmed HIV infections.^[12]^ While both studies provide valuable updates, the current study is distinct in its larger sample size (n = 49,296) and in being the first in Jordan to apply a stratified month-by-month χ^2^ trend analysis, enabling detection of seasonal patterns in TTI prevalence. Given the prolonged presence of Syrian refugees and shifting national policies on their health system access, Jordan faces unique public health and infectious disease surveillance challenges as one of the world’s major refugee-hosting countries.^[13]^

Although refugee-specific data are not available in this analysis, global evidence consistently shows that displaced populations face heightened vulnerability to infectious diseases due to overcrowding, limited access to healthcare, and low immunization coverage. This concern is underscored by the 2023 UNAIDS report, which documented 1.3 million new HIV infections globally, 44% of which were among women and girls, and highlighted that people in unstable or marginalized settings, including refugees, remain disproportionately affected.^[14]^ These conditions, especially when coupled with increased pressure on national health infrastructure, may influence infection dynamics in host populations. Therefore, renewed national surveillance is essential to address the absence of updated TTI data in refugee-hosting environments like Jordan. This study aims to provide an updated assessment of the prevalence of transfusion-transmitted infections among healthy blood donors in Jordan. The findings may support national transfusion safety efforts and inform future epidemiological monitoring and policy development. In doing so, it helps close the surveillance gap since 2016 and offers new insight into national blood safety, infection trends, and public health readiness amid demographic strain.

## 2. Materials and methods

### 2.1. Study design

This retrospective study was conducted between July 2018 and November 2019 at the Princess Iman Center for Research and Laboratory Sciences, in collaboration with the Department of Medical Laboratory Sciences at Al-Ahliyya Amman University, Amman, Jordan. The blood bank facility at this center operates under the Royal Medical Services (RMS) network and is responsible for collecting approximately 3000 blood units per month. It provides comprehensive hemotherapy services to all affiliated healthcare departments within King Hussein Medical City, located in Amman, Jordan.

The primary objective of the study was to screen whole blood donors for TTIs and to evaluate the prevalence of these infections within the RMS donor population. The study was carried out in coordination with the second-largest blood bank facility in the country, following the National Blood Bank Unit in Amman. Other national blood supply organizations, such as the Jordanian Red Crescent, were not involved in this study.

This time period was selected to capture seasonally distributed donor activity over a 17-month span, including both high- and low-demand donation periods. With 49,296 donations screened, this study represents the largest national dataset of its kind reported in Jordan since 2016, providing a valuable post-baseline update for TTI surveillance. The scale and continuity of this dataset offer a unique opportunity to assess both overall prevalence and intra-year variation in infection rates. This study represents the first known national effort to apply stratified monthly trend analysis (using χ^2^) to blood donor TTI data in Jordan, enhancing its utility for seasonal surveillance and policymaking.

### 2.2. Sampling

The blood bank facility, located in Amman the capital of Jordan coordinates blood collection and transfusion services for 12 hospitals and medical centers across the RMS network. It collects approximately 3000 units of blood per month. All accepted whole blood donors during the study period were included after receiving the necessary ethical and institutional approvals, totaling 49,296 recruited donation units. All donors met the inclusion criteria outlined by the Jordanian Ministry of Health, which required donors to be between 18 and 65 years of age, weigh more than 50 kg, have hemoglobin levels above 12.5 g/dL, and be free of acute or chronic illness. Donors also underwent a physical examination and completed a medical history evaluation to ensure the safety of the donation process. All donors participated as first-time donors; for frequent or regular donors, only the first donation within the study period was included to avoid duplication and ensure sample independence.

Only a single record was retained for donors who donated more than once during the study period. All donors in this study were Jordanian nationals, in accordance with the RMS’s blood donation regulations. This contrasts with the National Blood Bank Center, where individuals of other nationalities are also eligible to donate blood. As per RMS regulations, only individuals holding Jordanian nationality were eligible to donate during this study period; migrant donors were not included. This exclusion criterion introduces a potential selection bias, as it prevents assessment of TTI prevalence among non-Jordanian populations, such as refugees and migrant workers, who represent an important demographic segment in Jordan. The vast majority of blood donors in this study (approximately 99%) were voluntary non-remunerated first-time donors. Donations were made either voluntarily or to support a relative in need of a transfusion, such as in cases involving planned surgeries.

As part of the standard eligibility screening conducted at the donation clinic, physiological parameters such as pulse rate, body weight, and body temperature were routinely assessed. However, these variables were not captured in the final study dataset, as the research focused solely on the serological assessment of TTIs. A 5 mL whole blood sample was collected from each donor using a yellow-cap blood collection tube. Samples were centrifuged at 2000 relative centrifugal force for 10 minutes. The serum was then separated and stored at 4°C prior to analysis. To ensure confidentiality, all samples were coded numerically with no identifying donor information. The only demographic data collected was the governorate location of each donor.

### 2.3. Laboratory TTI screenings

The blood bank unit at Princess Iman Center employs 46 qualified technologists holding diplomas or bachelor’s degrees in laboratory sciences or nursing, in addition to staff with master’s degrees or MDs in Pathology or Transfusion Medicine. Certified nurses manage donor registration, phlebotomists conduct blood collection, and technologists perform processing and screening. All staff are trained, privileged, and evaluated by a Clinical Privileging Committee, with ongoing competency assessments to ensure high standards. Supervision by a qualified Medical Director ensures adherence to standard operating procedures, internal audits, and documentation, consistent with national and international transfusion safety regulations. The Roche Elecsys Cobas e601 (Roche Diagnostics, Germany) automated analyzer was used to screen donors’ serum samples for TTI. The system utilizes the Electrochemiluminescence Assay (ECLA) for detection of TTI using the following kits: HBsAg (Elecsys HBsAg II, Roche Diagnostics, Germany), HBcAb (Elecsys Anti-HBc II, Roche Diagnostics, Germany), HCV Ab (Elecsys anti-HCVII, Roche Diagnostics, Germany), and HIV 1/2 Ab (Elecsys HIV combi PT, Roche Diagnostics, Germany). For syphilis, rapid plasma reagin-carbon (Linear Chemicals, Spain) was used. Manufacturer’s instructions were followed for each testing procedure. The Roche Elecsys Cobas e601 is fully automated to minimize operator variability and is calibrated regularly by authorized company agents. Quality control is maintained using lyophilized blood-based controls and external run controls. Instrument calibration and routine internal quality controls were performed in accordance with the manufacturer’s recommendations to ensure assay accuracy and reliability.

Positive results were confirmed by repeating the sample in duplicate using another methodology as follow: Sandwich enzyme-linked immunosorbent assay (ELISA) with Monolisa HBsAg ULTRA (BIO-RAD, France) was used to confirm results for HBsAg marker. Anti-HCV V 4.0 (DIA Source Immunoassay, BioSource, Belgium) was used to confirm hepatitis C antibodies marker results. Human immunodeficiency virus antibody & Ag ELISA kit (DIA.PRO Diagnostic Bioprobes, Italy) was used to confirm positive HIV screening test. Anti-*Treponema palladium* antibodies-*Treponema pallidum* hemagglutination assay (Linear Chemicals, Spain) was used to confirm screening for TP-Ab positive results. Manufacturer’s instructions were followed for each testing procedure. Positive results detected by the Cobas e601 were confirmed by ELISA testing performed by different operators to reduce inter-rater bias. Any discrepancies were resolved by the Ministry of Health Central Laboratory through donor recall and medical evaluation. HIV 1/2 and syphilis reactive samples were sent to the Jordan Ministry of Health (JMOH) Central Public Health Laboratory, Amman, Jordan for confirmation according to the JMOH’s rules and regulations.

### 2.4. Data analysis

The distribution of blood donors across the various governorates of Jordan was analyzed and expressed as both absolute numbers and percentages of the total donor population included in the study. The prevalence of each transfusion-transmissible infection (TTI) was calculated as a percentage of the total donor cohort. Additionally, monthly distribution patterns of each TTI were assessed to evaluate trends in infection rates over the 17-month study period.

To identify potential seasonal or temporal variability in infection markers, a χ^2^ test for independence was applied to monthly prevalence data for each TTI. This statistical approach allowed for the detection of significant month-to-month differences, particularly for HBcAb and HBsAg, thus introducing a temporal dimension not previously explored in national TTI surveillance studies in Jordan.

All data were compiled using Microsoft Excel 2019 (Microsoft Corporation, Redmond) and stored in password-protected files to ensure data security. Statistical calculations, including prevalence rates and percentage distributions, were performed using the same software.

### 2.5. Ethical approval

This study was approved by the Institutional Review Board of the RMS under approval number 6/20, dated June 26, 2018. Supporting documentation is provided in the supplementary files.

In compliance with the Institutional Review Board approvals obtained, access to detailed socio-demographic information was restricted. The research team was not permitted to access personal identifiers or additional demographic variables beyond gender and donor governorate. Only anonymized data, including gender, location, and TTI screening results, were made available for analysis.

## 3. Result

This cross-sectional study was conducted over 17 months, from July 2018 to December 2019. A total of 49,296 blood units were collected from eligible donors at the King Hussein Medical Centre, a central facility within the Jordanian RMS network. Most of the donations (n = 48,460; 98.24%) came from residents of the Amman governorate, while only 1.76% of units were collected from other regions of Jordan. Donations were primarily made voluntarily or as family replacements. A total of 98.2% of the blood donors were male, while 1.8% were female. The majority of donated blood units were collected from donors residing in the Amman governorate, accounting for 98.24% of the total (Table 1). The remaining 1.82% of units were obtained from donors located in other Jordanian governorates. Most donations were made voluntarily or for family replacement purposes.

**Table 1:** Distribution of blood donors by governorate in Jordan (n = 49,269).

Governorate	Number of units (n)	Percentage (%)
Amman	48,400	98.24
Irbid	155	0.31
Ajloun	56	0.11
Jerash	86	0.17
Mafraq	62	0.13
Balqa	86	0.17
Zarqa	178	0.36
Madaba	113	0.23
Karak	74	0.15
Tafilah	31	0.06
Ma’an	25	0.05
Aqaba	30	0.06
Total	49,269	

All collected blood units were screened for TTIs, including HBsAg, HBcAb, HCV Ab, HIV 1/2 antibodies, and *Treponema pallidum* antibodies. Initial screening was performed using the Roche Elecsys Cobas e601 analyzer, and all reactive samples were retested using confirmatory ELISA kits.

**Figure 1.**
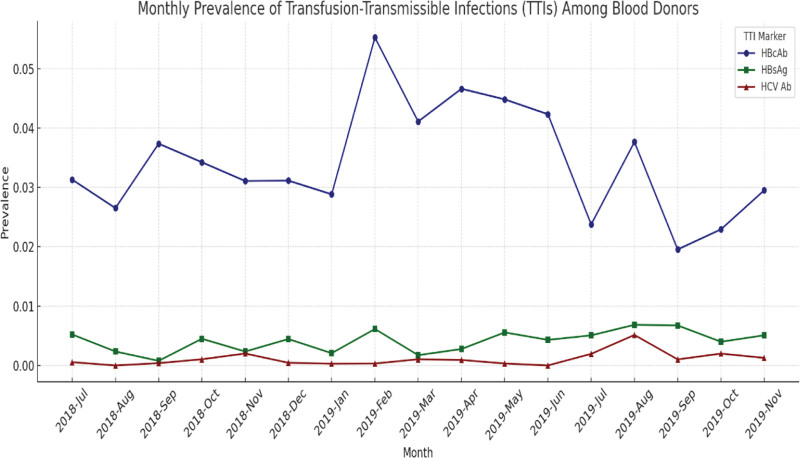
Monthly prevalence trends of HBcAb, HBsAg, and HCV Ab among blood donors in Jordan, July 2018 to November 2019. The line graph displays the monthly proportion of transfusion-transmissible infection markers: hepatitis B core antibody (HBcAb, blue circles), hepatitis B surface antigen (HBsAg, green squares), and hepatitis C virus antibody (HCV Ab, red triangles). HBcAb showed the highest and most variable prevalence, peaking in February 2019. HBsAg and HCV Ab levels remained consistently low throughout the study period. All symbols are defined in the legend. No arrows are used in the figure. HBcAb = hepatitis B core antibody, HBsAg = hepatitis B surface antigen, HCV Ab = hepatitis C virus antibody.

Out of the total sample, 1694 donors (3.44%; 95% confidence interval (CI): 3.28–3.61%) tested positive for HBcAb, and 202 donors (0.41%; 95% CI: 0.36–0.47%) were positive for HBsAg. HCV antibodies were confirmed in 51 donors (0.10%; 95% CI: 0.07–0.13%), while no confirmed positive results were observed for HIV or syphilis. Table 2 presents the monthly distribution of confirmed TTI-positive cases and total blood units screened across the 17-month study period. In addition, Figure 1 provides a graphical representation of monthly prevalence trends for HBcAb, HBsAg, and HCV Ab, complementing the tabular data and highlighting temporal fluctuations more clearly.

**Table 2:** Monthly number and percentage of positive TTI markers among blood donors in Jordan, July 2018 to November 2019 (n = 49,296).

Year	Month	HBcAb	HBsAg	HCV Ab	HIV	RPR	Total # of units screened
2018	July	114	19	2	0	0	3641
	August	79	7	0	0	0	2980
	September	97	2	1	0	0	2596
	October	99	13	3	0	0	2892
	November	93	7	6	0	0	2993
	December	70	10	1	0	0	2248
2019	January	98	7	1	0	0	3399
	February	171	19	1	0	0	3095
	March	119	5	3	0	0	2896
	April	152	9	3	0	0	3261
	May	137	17	1	0	0	3056
	June	118	12	0	0	0	2788
	July	61	13	5	0	0	2568
	August	66	12	9	0	0	1753
	September	58	20	3	0	0	2968
	October	69	12	6	0	0	3010
	November	93	16	4	0	0	3152
Total		1694	200	49	0	0	49,296
Percentage (%)		3.44	0.41	0.1	0	0	–

HBcAb = hepatitis B core antibody, HBsAg = hepatitis B surface antigen, HCV Ab = hepatitis C virus antibody, HIV = human immunodeficiency virus, RPR = rapid plasma regain, TTI = transfusion-transmissible infection.

This study is the first in Jordan to apply monthly stratification and χ^2^ testing to such a large dataset of blood donor TTI results. This analytical approach adds a novel temporal dimension to TTI surveillance and enables the detection of seasonally associated variation that may otherwise remain hidden in aggregate prevalence data.

Among the 1694 HBcAb-positive donors, 149 (8.8%) were also HBsAg-positive, indicating a possible chronic or active HBV infection. The remaining 1545 donors were HBcAb-positive but HBsAg-negative, suggesting past exposure or resolved infection. Additionally, 51 donors were HBsAg-positive and HBcAb-negative, bringing the total number of HBsAg-positive cases to 202.

After accounting for duplicate positives, the total number of blood units rejected due to HBV was 1747 (3.54%), calculated as follows: (1694 HBcAb-positive + 202 HBsAg-positive − 149 dual positives). These results are summarized in Table 3. Three samples were initially reactive for HIV 1/2 antibodies, but all tested negative upon confirmatory Western Blot analysis at the Jordan Ministry of Health Central Public Health Laboratory, indicating false positives. No confirmed cases of HIV or syphilis were detected in this study cohort, consistent with previous national data.

**Table 3:** HBV marker serostatus combinations (HBsAg and HBcAb) among blood donors in Jordan, 2018–2019.

Donors n (%)	HBsAg	HBcAb
149 (8.52%)	+	+
51 (2.919%)	+	−
1545 (88.4%)	−	+

HBcAb = hepatitis B core antibody, HBsAg = hepatitis B surface antigen.

To contextualize these findings, Table 4 presents a comparative summary of TTI prevalence rates reported in previous national studies conducted in Jordan.^[11,15]^ Notably, our study includes the largest single-site dataset since 2016, and is the first to implement stratified monthly χ^2^ analysis. While prevalence estimates for HBV and HCV markers in this study generally fall within the ranges reported in prior literature, the observed HBcAb positivity of 3.44% appears elevated relative to the national average (2.69–4.11%). In contrast, HBsAg and HCV Ab rates are slightly lower than those previously observed. These differences may reflect sampling variability, changing epidemiological patterns, or population characteristics specific to the RMS blood donor pool.

**Table 4:** Comparative prevalence of TTI markers among blood donors reported in published national studies from Jordan, including the current study (2018–2019).

Study	Year range	Sample size	HBsAg (%)	HBcAb (%)	HCV Ab (%)	HIV (%)	Syphilis (%)
Current study	2018–2019	49,296	0.41	3.44	0.1	0	0
Souan et al (2021)	2014–2019	88,565 (KHCC); 1,224,933 (national)	0.21–0.46	2.69–4.11	0.05–0.13	0	Not reported
Hroob et al (2020)	2013–2018	365,029	0.13 (F), 0.37 (M)	Not reported	0.07 (F), 0.12 (M)	0 (F), 0.005 (M)	0.0003 (F), 0.02 (M)

HBcAb = hepatitis B core antibody, HBsAg = hepatitis B surface antigen, HCV Ab = hepatitis C virus antibody, HIV = human immunodeficiency virus, TTI = transfusion-transmissible infection.

The prevalence of HBcAb, HBsAg, and HCV Ab was calculated alongside their 95% CIs to assess monthly variation in infection markers. These estimates help identify potential temporal fluctuations and provide statistical context for observed trends (Table 5).

**Table 5:** Monthly prevalence and 95% CI for HBcAb, HBsAg, and HCV Ab among blood donors in Jordan, July 2018 to November 2019 (n = 49,296).

Month	HBcAb prevalence	HBcAb CI lower	HBcAb CI upper	HBsAg prevalence	HBsAg CI lower	HBsAg CI upper	HCV Ab prevalence	HCV Ab CI lower	HCV Ab CI upper
July 2018	0.03131	0.025653	0.036967	0.005218	0.002878	0.007559	0.000549	−0.00021	0.00131
August 2018	0.02651	0.020742	0.032278	0.002349	0.000611	0.004087	0	0	0
September 2018	0.037365	0.030069	0.044661	0.00077	−0.0003	0.001838	0.000385	−0.00037	0.00114
October 2018	0.034232	0.027605	0.040859	0.004495	0.002057	0.006933	0.001037	−0.00014	0.002211
November 2018	0.031073	0.024856	0.037289	0.002339	0.000608	0.004069	0.002005	0.000402	0.003607
December 2018	0.031139	0.023959	0.038319	0.004448	0.001697	0.007199	0.000445	−0.00043	0.001317
January 2019	0.028832	0.023206	0.034458	0.002059	0.000535	0.003584	0.000294	−0.00028	0.000871
February 2019	0.05525	0.047201	0.0633	0.006139	0.003387	0.008891	0.000323	−0.00031	0.000956
March 2019	0.041091	0.033861	0.048321	0.001727	0.000214	0.003239	0.001036	−0.00014	0.002208
April 2019	0.046611	0.039376	0.053847	0.00276	0.000959	0.004561	0.00092	−0.00012	0.001961
May 2019	0.04483	0.037493	0.052167	0.005563	0.002926	0.0082	0.000327	−0.00031	0.000968
June 2019	0.042324	0.034851	0.049798	0.004304	0.001874	0.006734	0	0	0
July 2019	0.023754	0.017864	0.029644	0.005062	0.002317	0.007807	0.001947	0.000242	0.003652
August 2019	0.03765	0.028739	0.04656	0.006845	0.002986	0.010705	0.005134	0.001788	0.00848
September 2019	0.019542	0.014562	0.024522	0.006739	0.003795	0.009682	0.001011	−0.00013	0.002154
October 2019	0.022924	0.017577	0.02827	0.003987	0.001736	0.006238	0.001993	0.0004	0.003587
November 2019	0.029505	0.023598	0.035413	0.005076	0.002595	0.007557	0.001269	2.62E−05	0.002512

CI = confidence intervals, HBcAb = hepatitis B core antibody, HBcAb CI = hepatitis B core antibody – confidence interval, HBsAg = hepatitis B surface antigen, HBsAg CI = hepatitis B surface antigen – confidence interval, HCV Ab = hepatitis C virus antibody, HCV Ab CI = hepatitis C virus antibody – confidence interval.

**Figure 2.**
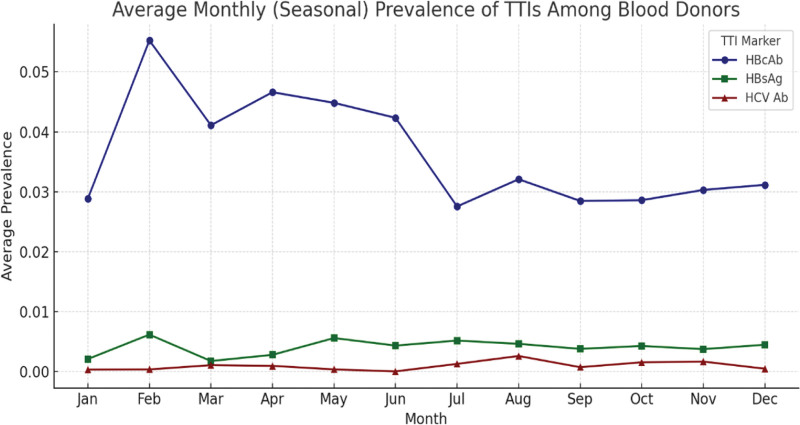
Seasonal distribution of average monthly prevalence of transfusion-transmissible infections among blood donors in Jordan. The plot displays average prevalence proportions of hepatitis B core antibody (HBcAb, blue circles), hepatitis B surface antigen (HBsAg, green squares), and hepatitis C virus antibody (HCV Ab, red triangles) across calendar months. HBcAb and HBsAg show seasonal clustering with notable peaks in February and late summer. HCV Ab remains consistently low. All markers and symbols are defined; no arrows appear in the figure. HBcAb = hepatitis B core antibody, HBsAg = hepatitis B surface antigen, HCV Ab = hepatitis C virus antibody

In addition to monthly trend visualization (Fig. 2), a plot was generated to assess average TTI marker prevalence by calendar month. This plot illustrates potential seasonal clustering of infections, notably a peak in HBcAb and HBsAg during February. While HCV Ab prevalence remained consistently low across months, the pattern for hepatitis B markers supports the findings of significant temporal variation detected in the χ^2^ analysis. The visualization offers further insight into potential behavioral, demographic, or campaign-related fluctuations in donor infection status.

Table 6 also forms the basis of a χ^2^ trend analysis, a novel method in Jordan’s TTI literature, used to test for statistically significant month-to-month variation in infection marker positivity. These tests provide deeper insight into potential underlying seasonal, behavioral, or campaign-driven donation patterns.

**Table 6:** Monthly distribution of positive and negative serological test results for HBcAb, HBsAg, and HCV Ab among blood donors in Jordan, July 2018 to November 2019.

Month	HBcAb_Pos	HBcAb_Neg	HBsAg_Pos	HBsAg_Neg	HCV_Pos	HCV_Neg	Total_Tested
July 2018	114	3527	19	3622	2	3639	3641
August 2018	79	2901	7	2973	0	2980	2980
September 2018	97	2499	2	2594	1	2595	2596
October 2018	99	2793	13	2879	3	2889	2892
November 2018	93	2900	7	2986	6	2987	2993
December 2018	70	2178	10	2238	1	2247	2248
January 2019	98	3301	7	3392	1	3398	3399
February 2019	171	2924	19	3076	1	3094	3095
March 2019	119	2777	5	2891	3	2893	2896
April 2019	152	3109	9	3252	3	3258	3261
May 2019	137	2919	17	3039	1	3055	3056
Jun 2019	118	2670	12	2776	0	2788	2788
July 2019	61	2507	13	2555	5	2563	2568
August 2019	66	1687	12	1741	9	1744	1753
September 2019	58	2910	20	2948	3	2965	2968
October 2019	69	2941	12	2998	6	3004	3010
November 2019	93	3059	16	3136	4	3148	3152

HBcAb_Neg = hepatitis B core antibody negative, HBcAb_Pos = hepatitis B core antibody positive, HBsAg_Neg = hepatitis B surface antigen negative, HBsAg_Pos = hepatitis B surface antigen positive, HCV_Neg = hepatitis C virus negative, HCV_Pos = hepatitis C virus positive.

To further assess monthly variability, raw counts of positive and negative cases for HBcAb, HBsAg, and HCV antibodies were compiled and analyzed (Table 5). These counts enabled the application of χ^2^ tests to explore the association between month and TTI positivity. Preliminary analysis suggested statistically significant month-to-month variation for HBcAb (χ^2^ = 98.71, *P* < .001) and HBsAg (χ^2^ = 42.85, *P* < .001), while no significant trend was found for HCV Ab (χ^2^ = 23.95, *P* = .059). Although not statistically significant, this result can be considered borderline, suggesting that with a larger sample size, potential temporal variation in HCV prevalence might become detectable. Future studies with expanded cohorts are therefore warranted to confirm this trend. This variability may be linked to seasonal, demographic, or institutional donation trends and warrants further investigation.

## 4. Discussion

This study offers the most recent update on the prevalence of transfusion-transmitted infections (TTIs) among blood donors in Jordan and highlights the evolving epidemiological landscape in the country. The findings reinforce the effectiveness of existing national blood safety measures while emphasizing the continued need for surveillance in light of demographic shifts and public health challenges. The overall prevalence rates for TTIs in this study were relatively low, with HBcAb at 3.44%, HBsAg at 0.41%, and HCV Ab at 0.10%, while no confirmed cases of HIV or syphilis were detected. This study provides an updated large-scale assessment of TTI prevalence in Jordan, making it a critical contribution to national blood safety data since 2016. Its expanded dataset (49,296 donations over 17 months) offers more robust statistical confidence and temporal insight compared to earlier studies.

The geographic distribution of donors (Table 1) raises concerns about the representativeness of the data. With over 98% of donations collected from Amman, the results may not accurately reflect infection patterns in more rural or underserved areas. Although Amman houses a large portion of Jordan’s population, these data may still skew national estimates. Broader sampling across diverse regions is needed to provide a comprehensive national assessment. Temporal patterns in infection rates were also explored (Table 2). While there was no clear seasonal trend, certain months such as February and September showed peaks in HBcAb and HBsAg positivity. These variations could be attributed to fluctuations in donor demographics, donation campaigns, or underlying behavioral trends, warranting further targeted investigation in future studies. This study’s incorporation of χ^2^ tests to assess monthly variation is a novel contribution to Jordan’s TTI literature. It provides early evidence of statistically significant temporal fluctuations in HBcAb and HBsAg prevalence, a dimension not previously analyzed in national surveillance.

To quantify this variability, monthly prevalence estimates and 95% CIs were calculated for each TTI marker (Table 4), revealing notable fluctuations in HBcAb and HBsAg positivity across the study period. Furthermore, χ^2^ analysis of raw seropositive and seronegative donor counts confirmed statistically significant month-to-month variation in HBcAb and HBsAg rates, while HCV showed no significant pattern (Table 5). In terms of specific serological markers, HBV indicators showed the most significant prevalence. The high proportion of HBcAb-positive but HBsAg-negative donors may indicate past resolved infections or occult HBV. Previous studies reported higher HBcAb rates (up to 6.04%) and HBsAg rates (0.52%) between 2009 and 2013, suggesting a downward trend that may be attributable to national vaccination initiatives, heightened awareness, and improved screening protocols.^[10]^

Compared to earlier studies, our findings show a declining trend in HBcAb and HBsAg prevalence, along with a slightly lower HCV rate. No confirmed HIV or syphilis cases were found, consistent with national data.^[11,15]^ Although this study did not assess ABO blood group associations, recent evidence suggests that blood group B donors in Nigeria may have a higher risk of HCV infection (1.6% overall prevalence; *P* < .02799), highlighting a potential area for future investigation in Jordanian donor populations.^[16]^ In addition, a study from Saudi Arabia reported O positive as the most common group (42%) and a low overall TTI prevalence of 1.002%, with anti-HBc as the most frequent marker, underscoring the value of investigating these associations in Jordan.^[17]^ Another study from Saudi Arabia found that O blood group donors were at higher risk of TTIs, with Kell blood group also showing significant associations with HBcAb, HIV, and syphilis, underscoring the importance of assessing blood group–infection dynamics in Jordan.^[18]^ These differences may reflect our younger, urban, and possibly healthier donor pool, mostly from Amman.^[11,12]^ Methodologically, this study is the first in Jordan to apply monthly stratified χ^2^ analysis, adding a temporal lens to TTI surveillance. This approach enhances policy relevance by identifying seasonal trends and offering a framework for real-time monitoring, especially important in the context of refugee-related demographic shifts.

Compared to the earlier national report, this study reveals both improved seroprevalence levels and enhanced analytical resolution, offering a stronger foundation for epidemiological inference. Further analysis of HBV markers (Table 3) revealed that 149 donors tested positive for both HBcAb and HBsAg, suggesting potential chronic or active infection. Another 51 were positive for HBsAg alone, while 1545 were HBcAb-only positive. Notably, 2 donors were also seropositive for HCV Ab in addition to HBsAg and HBcAb, indicating co-infection with hepatitis B and C viruses.

The present study found HBV prevalence rates of 0.41% for HBsAg and 3.44% for HBcAb among 49,269 Jordanian donors. More recent national data similarly report HBsAg rates ranging from 0.18 to 0.34% and anti-HBc rates between 2.68% and 4.11% among Jordanian blood donors between 2014 and 2019, indicating sustained low prevalence with a slight increase observed in 2019.^[15]^ In contrast, a study among pregnant women in Jordan documented higher HBsAg prevalence of 5%, reflecting differences in population risk profiles.^[19]^ Regionally, HBV prevalence remains considerably higher, such as in Somalia, where 9.7% of blood donors tested positive for HBsAg, underscoring Jordan’s relatively lower endemicity.^[20]^

The absence of HIV and syphilis in confirmed samples is consistent with earlier data and reflects both the low prevalence of these infections in the general population and the effectiveness of current donor screening strategies. However, this should not be viewed as a reason for complacency. Cultural stigma around HIV and other sexually transmitted infections in Jordan may result in underreporting and undiagnosed cases, reinforcing the importance of anonymous, population-based testing and awareness programs.^[21,22]^ Recent evidence shows that relying on HBcAb-positive but HBsAg-negative donors carries residual risk of occult HBV infection. For example, in Sokoto, Nigeria, HBV deoxyribonucleic acid (DNA) was detected in 1.5% of HBcAb-positive donors despite being HBsAg-negative, confirming the potential for transmission if nucleic acid testing (NAT) is not performed.^[23]^ Similarly, in Jiangsu Province, China, electrochemiluminescence immunoassay detected HBV markers in 43.15% of previously reactive donors compared with only 4.75% by ELISA and 3.12% by NAT, underscoring that standard screening alone may miss occult carriers.^[24]^ These findings suggest that blood from HBcAb-positive but HBsAg-negative donors can only be considered safe when supplemented with sensitive assays such as NAT or HBV DNA testing.

Reevaluating these deferral policies could mitigate unnecessary donor loss, especially in low-endemicity contexts like Jordan. In contrast, current Jordanian guidelines adopt a more conservative approach, deferring HBcAb-positive donors regardless of HBsAg status to minimize transfusion risk. The high prevalence of HBcAb relative to HBsAg observed in this study likely reflects past resolved infections or low-level chronic carriers. This study also draws attention to the limitations of data representativeness, with 98.2% of donations collected from Amman, potentially skewing national estimates. Given that 85% of Jordan’s population resides in Amman, these results may still reflect general population trends. However, more geographically diverse sampling is essential to capture the full picture, especially in governorates with different socioeconomic and health profiles.

The prolonged presence of Syrian refugees and evolving national health policies further complicate the interpretation of infection trends, as displaced populations often originate from regions with higher endemicity of TTIs, adding to Jordan’s surveillance burden. Although this study did not distinguish between citizen and refugee donors, the broader public health implications are clear. Displaced populations often face barriers to healthcare access and immunization, increasing their vulnerability to infectious diseases. Thus, the findings of this study highlight the necessity of inclusive and disaggregated TTI surveillance, in line with SDG 3.3, which aims to end the epidemics of HIV, hepatitis, and other communicable diseases by 2030.^[9,25]^ As a host country with one of the highest refugee-to-population ratios globally, Jordan must integrate TTI monitoring into broader disease surveillance systems. This study demonstrates how blood donor data can serve as a proxy for real-time epidemiological surveillance in refugee-hosting settings. These findings contribute to evidence-based planning aligned with WHO’s blood safety strategy and SDG 3.3 goals to eliminate major communicable diseases by 2030.

While the declining trend in TTIs is encouraging, continued monitoring is critical to anticipate and manage emerging threats. National policies should leverage this large-scale dataset as a benchmark for future trend tracking and intervention design. Future studies should explore the utility of molecular testing (e.g., NAT for HBV DNA and HCV ribonucleic acid) and longitudinal surveillance models to identify and prevent TTI transmission more effectively. Additionally, investing in algorithmic modeling tools may enable policymakers to predict TTI trends and respond preemptively with targeted interventions. This study provides a comprehensive and updated assessment of transfusion-transmitted infections (TTIs) among blood donors in Jordan, revealing low prevalence rates for hepatitis B and C, and no confirmed cases of HIV or syphilis. These findings reflect progress in public health interventions such as vaccination, awareness programs, and robust screening protocols, aligning with WHO’s goal of eliminating major infectious diseases by 2030. This dataset offers a timely surveillance benchmark that directly supports Jordan’s public health agenda and aligns with ongoing national monitoring efforts. By applying monthly temporal analysis, it provides new insight into fluctuations in infection trends, which is critical for proactive monitoring and seasonal risk assessment.

The study affirms the value of blood donor surveillance as a proxy for tracking infection trends in the general population, especially amid ongoing regional demographic changes. Its methodological scope, including χ^2^-based trend detection, enhances its policy relevance by informing evidence-based decisions on screening thresholds and donor management in both routine and emergency health settings. This study serves as a benchmark for integrating seasonal surveillance models into Jordan’s national blood safety strategy, offering a scalable framework for evidence-based policy planning. Its findings complement global health objectives, particularly the WHO’s blood safety strategy and the United Nations’ SDG 3.3 target of eliminating major communicable diseases by 2030.

However, the study has 2 notable limitations. Although the dataset predates the COVID-19 pandemic, it remains the most comprehensive post-2016 national dataset for TTI surveillance. First, the data is geographically skewed, with over 98% of samples collected from the Amman governorate, limiting national generalizability. Donor data are limited to the Amman governorate; generalizability is constrained, but Amman houses the majority of the population and remains a critical urban surveillance point. Second, restricting eligibility to Jordanian nationals introduces a potential selection bias, as it excludes refugees and migrant populations who constitute a substantial demographic in Jordan and may exhibit different infection epidemiology. Third, the lack of data on refugee status prevents an assessment of how this high-risk subgroup may influence national TTI trends. Finally, physiological parameters routinely assessed during donor screening (e.g., pulse rate, body weight, body temperature) were not captured in the final dataset, which prevented analysis of their potential relationship with TTI prevalence. However, as the study’s primary focus was serological surveillance, this impact is likely limited.

For future work, national efforts should focus on expanding surveillance to include donors from underrepresented regions, integrating molecular testing (e.g., NAT and HBV DNA) to improve diagnostic accuracy, and developing algorithmic tools to predict transmission trends and inform preventive strategies. Continued investment in high-resolution, nationwide blood safety monitoring, integrated with demographic risk mapping, will be essential to achieving both national and global infectious disease elimination targets.

## 5. Conclusion

This study provides an updated assessment of TTIs among blood donors in Jordan, offering clinically relevant insights for national blood safety. The findings demonstrate low prevalence rates for hepatitis B and C, with no confirmed cases of HIV or syphilis, underscoring the effectiveness of current donor screening protocols.

The application of temporal trend analysis highlights fluctuations in HBV markers, which has direct implications for refining surveillance strategies and optimizing donor management policies. Clinically, these results reinforce the value of blood donor data as a reliable proxy for monitoring infection dynamics within the wider population, particularly in a country hosting large displaced communities. By strengthening the evidence base for safe transfusion practices, this study supports ongoing national and global efforts to ensure the availability of safe blood and aligns with WHO targets for the elimination of major communicable diseases.

## Author contributions

**Conceptualization:** Ali Atoom, Sanaa Al-Saeidat, Talal Al-Qaisi.

**Data curation:** Ali Atoom, Baheieh Al-Abadi, Mansour Al-Hiary, Sanaa Al-Saeidat.

**Formal analysis:** Ali Atoom, Talal Al-Qaisi.

**Investigation:** Ali Atoom, Baheieh Al-abadi.

**Methodology:** Ali Atoom, Bayan Alzubi, Sanaa Al-Saeidat.

**Project administration:** Ali Atoom, Baheieh Al-Abadi, Talal Al-Qaisi.

**Resources:** Ali Atoom, Baheieh Al-abadi, Mansour Al-Hiary, Bayan Alzubi, Sanaa Al-Saeidat.

**Software:** Ali Atoom, Mansour Al-Hiary, Bayan Alzubi.

**Supervision:** Ali Atoom.

**Validation:** Ali Atoom, Talal Al-Qaisi.

**Visualization:** Ali Atoom.

**Writing – original draft:** Ali Atoom.

**Writing – review & editing:** Ali Atoom, Baheieh Al-Abadi, Mansour Al-Hiary, Bayan Alzubi, Sanaa Al-Saeidat, Talal Al-Qaisi.
